# A macrocyclic molecule with multiple antioxidative activities protects the lens from oxidative damage

**DOI:** 10.3389/fchem.2022.996604

**Published:** 2022-10-28

**Authors:** Jinmin Zhang, Yu Yu, Magy A. Mekhail, Hongli Wu, Kayla N. Green

**Affiliations:** ^1^ Pharmaceutical Sciences, College of Pharmacy, University of North Texas Health Science Center, Fort Worth, TX, United States; ^2^ Department of Chemistry and Biochemistry, Texas Christian University, Fort Worth, TX, United States; ^3^ North Texas Eye Research Institute, University of North Texas Health Science Center, Fort Worth, TX, United States

**Keywords:** ^OH^Py_2_N_2_, lens, oxidative stress, Nrf2, glutaredoxin, ferroptosis

## Abstract

Growing evidence links oxidative stress to the development of a cataract and other diseases of the eye. Treatments for lens-derived diseases are still elusive outside of the standard surgical interventions, which still carry risks today. Therefore, a potential drug molecule ^OH^Py_2_N_2_ was explored for the ability to target multiple components of oxidative stress in the lens to prevent cataract formation. Several pathways were identified. Here we show that the ^OH^Py_2_N_2_ molecule activates innate catalytic mechanisms in primary lens epithelial cells to prevent damage induced by oxidative stress. This protection was linked to the upregulation of Nuclear factor erythroid-2-related factor 2 and downstream antioxidant enzyme for glutathione-dependent glutaredoxins, based on Western Blot methods. The anti-ferroptotic potential was established by showing that ^OH^Py_2_N_2_ increases levels of glutathione peroxidase, decreases lipid peroxidation, and readily binds iron (II) and (III). The bioenergetics pathway, which has been shown to be negatively impacted in many diseases involving oxidative stress, was also enhanced as evidence by increased levels of Adenosine triphosphate product when the lens epithelial cells were co-incubated with ^OH^Py_2_N_2_. Lastly, ^OH^Py_2_N_2_ was also found to prevent oxidative stress-induced lens opacity in an *ex vivo* organ culture model. Overall, these results show that there are multiple pathways that the ^OH^Py_2_N_2_ has the ability to impact to promote natural mechanisms within cells to protect against chronic oxidative stress in the eye.

## Introduction

Cataracts are the most common cause of vision loss in individuals over the age of 60 as well as the leading cause of blindness in the world. According to the latest assessment from the World Health Organization, more than half of the cases of blindness worldwide are attributed to cataracts (Organization, 2019). Moreover, the trend toward an increased aging population has built global attention to reducing the prevalence of cataract. Currently, the only treatment option to correct a cataract patient’s vision is surgery. Though cataract surgery is a well-established procedure, complications may still arise, especially in patients of advanced age. Surgery-related complications such as posterior capsular opacity, intraocular lens dislocation, eye inflammation, photopsia, ocular hypertension, and macular edema may cause irreversible blindness (Chan et al., 2010). Therefore, there is still a great need to identify non-surgical therapeutic options with benefits outweighing the risks of surgery.

Oxidative stress encompasses a range of deficiencies in the balance between pro-oxidant and antioxidant regulatory pathways and the related damage to biological structures (Naik et al., 2014; Czerska et al., 2015). The eye is a prominent target of oxidative stress. It is continuously exposed to various oxidative conditions, such as photo-oxidation, ultraviolet (UV) radiation, smoke, and various forms of pollutants. Thus, oxidative stress has been associated with many ocular disorders, notably age-related macular degeneration (AMD) and cataracts (Spector, 1995; Beatty et al., 2000; Lou, 2003; Yildirim et al., 2011). Chronic exposure to light from the Sun, the largest source of UV radiation in our environment, can significantly increase the risk of a cataract, particularly if the recipient is over 40 years of age (Wolff, 1994). Solar UV that penetrates the atmosphere is mainly composed of UVA (320–400 nm) and UVB (290–320 nm) radiation (Zigman, 1995). This strong UV energy induces overproduction of reactive oxygen species (ROS) that damage lens epithelial cells (LECs), disturb the energy metabolism pathways, promote insoluble protein aggregation, and eventually lead to lens opacity (Lofgren, 2017). Therefore, the need to rebalance ROS to prevent chronic disease like this in the eye continues to be a driving force for the development of new, targeting molecules and studies to understand the mechanisms of their action(s).

Creating interventions for uncontrolled ROS is challenging, but there is strong evidence that antioxidant approaches to prevent or curb disease have positive impacts and show this is a feasible approach (Pisoschi et al., 2021; Woo et al., 2014; Bosco et al., 2011; DeCensi et al., 2010; Goodson et al., 2009; Zhang, 2019; D'Souza et al., 2020; Nechuta et al., 2011; Sotgia et al., 2011; Giem et al., 1993; Fraser, 2009; Singh et al., 2003; Cooper et al., 2009; Zhang et al., 2014a; Farrawell et al., 2018; Rowe et al., 2020; Ikawa et al., 2015; Williams et al., 2016). For example, the Age Related Eye Disease Study sponsored by the National Eye Institute strongly support that high levels of antioxidant vitamins and zinc can reduce the risk of advanced AMD and its associated vision loss (Age-Related Eye Disease Study Research Group, 2001; Chew et al., 2013). Nevertheless, there remains a gap between the current understanding of oxidative stress in disease pathologies and the development of new therapeutic molecules that successfully target this etiology (Lannfelt et al., 2008; Alzheimer’s Disease Facts, 2016; Jarvis, 2015; Ratner, 2015; Niedzielska et al., 2016; Doig et al., 2017; Kingwell, 2017; Crielaard et al., 2017; Patel, 2016). During drug design for a disease, therapeutics are typically targeted toward one causative agent ex: cholesterol, an enzyme, or gene. However, such an approach is complicated for diseases involving oxidative stress because the etiology of oxidative stress is multifactorial. Identification of a therapeutic agent that can target multiple antioxidant pathways or induce defense mechanisms innate to the cell could prove powerful in fighting these diseases.

**FIGURE 1:**
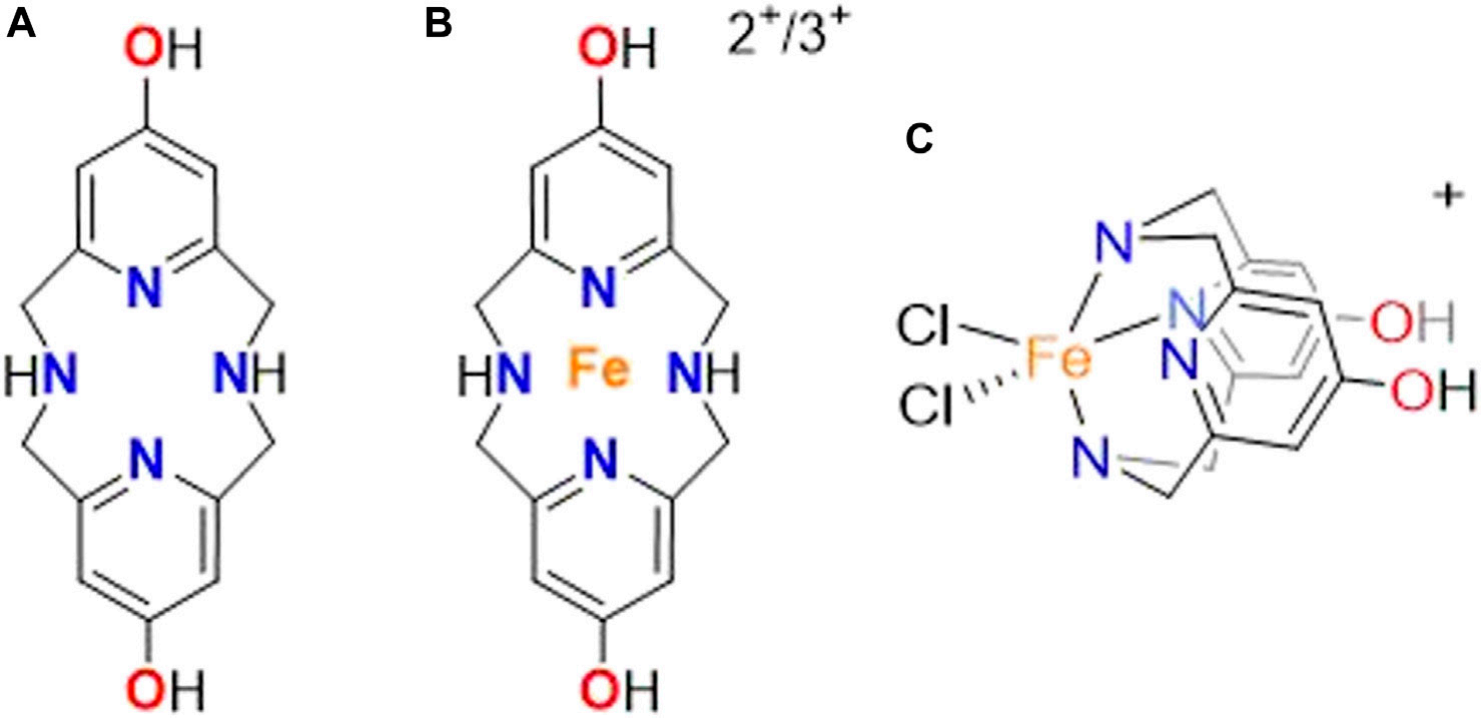
Structure of the **(A)** ^OH^Py_2_N_2_ molecule along with the connectivity of the **(B)** iron (II) and (III) complex and **(C)** orientation of the iron in the ^OH^Py_2_N_2_ ligand based on UV-vis studies.

As a result of the current challenges for diseases that involve oxidative stress, we designed the ^OH^Py_2_N_2_ multimodal small molecule (Figure 1) with direct targeting reactivity against ROS (Johnston et al., 2019). Several benchtop assays validated our initial hypothesis that the water soluble molecule could function as a direct antioxidant molecule. Integration of a hydroxyl substituted pyridine group within a tetra-aza macrocyclic ring generates the ^OH^Py_2_N_2_ small molecule that can readily scavenge free radicals as well as bind mis-regulated transition metal ions, halting toxic metal redox processes (Green et al., 2019; Johnston et al., 2019). Interestingly and not part of our initial design, which focused on direct reactivity with ROS, ^OH^Py_2_N_2_ showed the potential to activate cellular antioxidant defense capacity *via* activation of the Nrf2 (Nuclear factor erythroid 2-related factor 2) signaling pathway in neuronal cell culture along with low toxicity, high metabolic stability, and proving to be highly water soluble (Johnston et al., 2019). Nrf2 is a transcription factor that plays key roles in antioxidant and detoxification responses, particularly in the eye. Nrf2 activates more than 250 antioxidant enzymes and pro-survival genes (Ma, 2013). It is commonly referred to as the gatekeeper to coordinated induction of cytoprotective and antioxidant genes or master antioxidant transcriptional regulator (Kaspar et al., 2009). Increased Nrf2 activity correlates with cellular survival and protection from oxidative injury to RPE cells and pathogenic processes (Johnson et al., 2009; Zhang et al., 2014b; Cuadrado et al., 2018; Tang et al., 2018; Bahn et al., 2019; Han et al., 2019; Kitaoka et al., 2019; Sotolongo et al., 2020). Likewise, lower Nrf2 expression is associated with an increased risk or early onset of diseases of the eye (Zhao et al., 2011; von Otter et al., 2010). Therefore, activation of Nrf2 pathways has garnered significant interest as a potential target for therapeutic design. The ability for ^OH^Py_2_N_2_ to activate this pathway piqued our interest to further explore the catalytic (enzyme based) and cellular pathways that this molecule might unlock to provide protection against oxidative stress.

The recently established understanding of the direct antioxidant activity of ^OH^Py_2_N_2_ through radical and transition metal scavenging, along with preliminary results suggesting catalytic protection *via* the Nrf2 pathway prompted the present studies focused on the therapeutic potential and activation of defensive antioxidant mechanisms induced by ^OH^Py_2_N_2_ to protect against oxidative stress in the eye (Johnston et al., 2019). To explore this potential, human lens epithelial cells were exposed to conditions that model oxidative stress and showed excellent cell viability when treated with ^OH^Py_2_N_2_. The levels of Nrf2 and downstream antioxidant enzyme GSH-dependent glutaredoxins (Grx1 and Grx2), important components that protect protein thiols from oxidation, increased ATP expression, and antiferroptotic potential were then measured to elucidate the catalytic protective mechanisms initiated by ^OH^Py_2_N_2_ treatment. Lastly, ^OH^Py_2_N_2_ was also found to prevent oxidative stress-induced lens opacity in an *ex vivo* organ culture model.

## Materials and methods

### General methods and materials

Ligand ^OH^Py_2_N_2_ was produced as the trihydrochloride salt using previously published methods (Johnston et al., 2019). Dulbecco’s modified Eagle’s medium (DMEM), fetal bovine serum (FBS), gentamicin, penicillin, and 0.05% trypsin were all purchased from Thermo Fisher Scientific (Waltham, MA, United States). Hydrogen peroxide and all chemicals were obtained from Sigma Chemical Co. (St. Louis, MO, United States) unless otherwise stated. Antibodies against Grx1 (ab45953) and Grx2 (ab45953) were purchased from Abcam (Waltham, MA, United States). Nrf2 (#12721) and GPX4 (#52455) antibodies were purchased from Cell Signaling (Denver, CO, United States). CellROX Deep Red was purchased from Thermo Fisher Scientific (Waltham, MA, United States). Colorimetric cell viability kit I (WST-8) was purchased from PromoKine (Heidelberg, Baden-Württemberg, Germany).

### Potentiometric methods

The concentration of the ^OH^Py_2_N_2_ ligand, as well as an estimation of the stability constant of the iron (II) complex, were determined *via* pH-potentiometric titrations. Challenges with solubility prevented a high level of accuracy in the models. A Metrohm 888 Titrando equipped with a Metrohm 6.0234.100 combined electrode was used to measure the pH in the titration experiments. For the calibration of the electrode, KH-phthalate (pH 4.008) and borax (pH 9.177) buffers were used (Manov et al., 1945; Manov et al., 1946). The calculation of [H^+^] from the measured pH values was performed with the use of the method proposed by Irving *et al.* by titrating a 0.02 M HCl solution with a standardized NaOH solution (0.2 M) (Irving et al., 1967). The differences between the measured and calculated pH values were used to obtain the [H^+^] concentrations from the pH-data collected in the titrations. The ion product of water was determined from the same experiment in the pH range 11.40–12.00. The ionic strength in the titrated and thermostat controlled (at 25°C) samples of 6.00 ml was kept constant and set to 0.15 M NaCl. The samples were stirred by a magnetic stir bar and kept under inert gas atmosphere (N_2_) to avoid the effect of CO_2_. The protonation constants of the ligands were obtained from previously reported data (Johnston et al., 2019). Validation of the formation of the metal complex was determined using the direct pH-potentiometric method by titrating samples with 1:1 metal-to-ligand ratios (the number of data pairs were between 100 and 250), allowing 1 min for sample equilibration to occur. Models of the formation were obtained from the titration data with the PSEQUAD program (Bush, 2011). The protonation and metal complex stability pH titration were plotted against the first derivative of the slope to emphasis the formation of the iron (II) complex of the ^OH^Py_2_N_2_.

### Primary mouse lens epithelial cell (LEC) culture

Primary LEC cultures were isolated from C57BL/6J mice. Mouse lens capsules were cut into small pieces and treated with 0.05% trypsin at 37°C for 10–20 min. The cells loosened from the capsule were placed into a 24-well plate containing 1 ml DMEM with 20% FBS and 50 μg/ml gentamicin per well. The cultures were incubated for 1 week in a humid atmosphere with 5% CO_2_ at 37°C. Medium was changed every 2 days. After the primary cultures achieved confluence, the cells were subcultured by using 0.05% trypsin.

### Lens organ culture

All experiments using mice were in accordance with the ARVO Statement for the Use of Animals in Ophthalmic and Vision Research and an approved institutional animal care and use committee protocol at the University of North Texas Health Science Center (Protocol Number: IACUC-2019-0003 and IACUC-2022-0008). The mouse lenses from C57BL/6J mice were carefully dissected out of the eye using a posterior approach to avoid the surgical tools damaging the lens. The lenses were cultured in modified TC-199 media by following previously published method (Cui and Lou, 1993). Briefly, the lenses were placed in a 24-well plate of TC-199 media with an osmolarity ranging from 300–320 mOsm. The medium was pre-incubated in an organ culture incubator at 37°C with 5% CO_2_ for 2 h prior to the addition of the lenses. The lenses were then incubated in the medium for 24 h. Those lenses became opaque, an indication of potential damage due to dissection, were excluded from the following experiments. Cultured lenses were incubated with 10–100 μL ^OH^Py_2_N_2_ for 24 h and then exposed to 200 μm H_2_O_2_ for another 6 h. The lens morphological changes were recorded under a dissecting microscope (Nikon) using darkfield illuminations. Lenses incubated with media alone served as the control.

### Cell viability assay

Cell viability was measured by a colorimetric cell viability kit (Promokine, Heidelberg, Germany) with the tetrazolium salt WST-8 (2-(2-methoxy-4-nitrophenyl)-3-(4-nitrophenyl)-5-(2,4-disulfophenyl)-2H-tetrazolium, monosodium salt), which can be bioreduced to a water-soluble orange formazan dye by dehydrogenases present in the viable cells. The amount of formazan produced is directly proportional to the number of living cells. Cells were seeded at a density of 5000 cells/well (100 μL total volume/well) in a 96-well assay. Cells were incubated with or without ^OH^Py_2_N_2_ (10 and 100 μm) for 24 h and then treated with 200 µm of H_2_O_2_ for 6 h. After treatment, 10 µL of WST-8 solution was added to each well of the culture plate and incubated for 2 h in the incubator. The absorption was evaluated at 450 nm using a microplate reader (BioTek, Winooski, VT). The cell morphology was observed under phase-contrast microscopy.

### Cellular ROS assay

Intracellular ROS levels were determined using the fluorescent probe CellROX Orange Reagent. After pretreatment with ^OH^Py_2_N_2_ for 24 h, LECs were exposed to 200 μm H_2_O_2_ for another 30 min. After treatment, the cells were loaded with 5 mm CellROX Orange Reagent for 30 min at 37°C, washed with DPBS and immediately imaged on a fluorescence microscope (Olympus, Center Valley, PA). The cellular fluorescence was quantified using the ImageJ software, after background subtraction for each image.

### Western blot analysis

Protein concentration was determined with a BCA assay kit (Thermo Scientific, Rockford, IL). Equal amounts of protein were boiled in Laemmli buffer (Bio-Rad, Bio-Rad, Richmond, CA, United States) and loaded onto 12% SDS-PAGE gel and transferred to a 0.2 µm polyvinylidene difluoride membrane (GE Healthcare, Boulder, CO). The membranes were incubated with appropriate primary antibodies for overnight at 4°C and were then incubated with the appropriate secondary antibodies for 1 h. Detection was performed using the ECL Western blotting detection system (Thermo Scientific, Rockford, IL). The immunoblot was analyzed with a Bio-Rad imaging system (Versadoc 5000 MP Imaging System, Bio-Rad, Richmond, CA, United States).

### Glutaredoxin activity

The enzyme activities of Grx1 and Grx2 were examined using 2-Hydroxyethyl disulfide (HEDS) as the substrate according to the method described in Raghavachari and Lou (Gladyshev et al., 2001). In brief, the cell lysate was mixed with potassium phosphate buffer (200 mm, pH7.5) containing 0.5 mm glutathione (GSH), 0.4 U/mL glutathione reductase (GR), 0.2 mm β-nicotinamide adenine dinucleotide phosphate, reduced tetra (cyclohexylammonium) salt (NADPH), and 2 mm hydroxyethyl disulfide (HED). The reaction was carried out at 30°C, and the decrease in absorbance at 340 nm was monitored for 5 min and used to determine the activity.

### ATP Quantification

ATP level was measured with an ATP bioluminescence assay kit CLS II (Roche Applied Science, Penzberg, Germany) according to the manufacturer’s recommendation (Wu et al., 2014). Briefly, lens homogenates were mixed with nine volumes of boiling solution (PBS containing 100 mm Tris and 4 mm EDTA) and incubated for 2 min at 100°C. The sample mix was then centrifuged at 1000 × g for 1 min, and 50 μL of the supernatant was mixed with 50 μL of luciferase reagent by automated injection. The luminescence intensity was detected by a Fluostar Optima microplate reader (BMG Labtech, Offenburg, Germany), and integrated for 1–10 s.

### Malondialdehyde (MDA) measurement

Cells were seeded at a density of 10,000 cells/well in a 96-well assay. Cells were incubated with or without ^OH^Py_2_N_2_ (1, 10 and 100 µm) for 24 h and then treated with 200 µm of H_2_O_2_ for 6 h. MDA was measured by using the MDA assay kit (Abcam, Cambridge, MA, United States, ab118970) based on the thiobarbituric acid (TBA) reaction. The intensity of the resulting MDA-TBA adduct was read at RFU at Ex/Em = 532/553 nm and the data were expressed as nmol/mg protein.

### Statistics

Each experiment was performed at least three times and statistical analyses were performed using Student’s t-test when comparing between two groups and one-way ANOVA followed by Bonferroni’s test as a post hoc test when comparing among three or more groups with the Prism software (GraphPad, La Jolla, CA). The number of experimental samples used in each group is presented in the figure legends. All data are expressed as means ± SD and differences were considered significant at *p* < 0.05.

## Results and discussion

Previous work has shown that pyridinophane type molecules are low molecular weight entities that display potent antioxidant ability, low toxicity, and high metabolic stability in neuronal cell culture models, suggesting they could serve as potential agents to treat Alzheimer’s and other neurodegenerative diseases (Lincoln et al., 2013; Green et al., 2019; Johnston et al., 2019). Among these molecules, the ^OH^Py_2_N_2_ pyridinophane (Figure 1) showed higher pharmacological efficacy and increased biological compatibility compared to the parent systems (Johnston et al., 2019). As a result of this success, disorders of the eye were next explored to understand the extent to which the protective nature of ^OH^Py_2_N_2_ could be utilized and as models to understand the mechanisms of this protective reactivity.

### ^OH^Py_2_N_2_ prevents LECs from H_2_O_2_-induced damage

**FIGURE 2:**
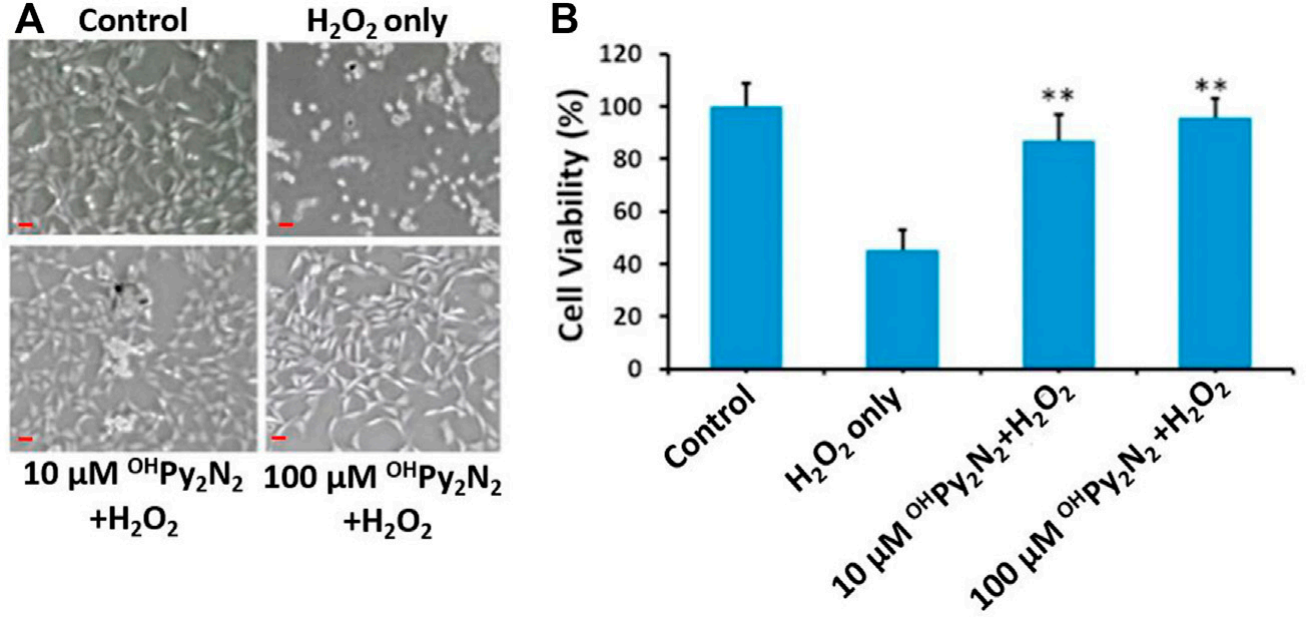
Cytoprotective effects of ^OH^Py_2_N_2_ in primary LEC against treatment with H_2_O_2_. Cells were incubated with or without ^OH^Py_2_N_2_ (10 and 100 µM) for 24 h and then treated with 200 µM of H_2_O_2_ for 6 h. After treatment, 10 µL of WST-8 solution was added to each well of the culture plate and incubated for 2 h in the incubator. **(A)** Images of cells (Scale bar = 50 µm), **(B)** Cell viability. *n* = 6, **p < 0.01.

To test the cytoprotective effects in the lens, primary LECs were treated with ^OH^Py_2_N_2_ for 24 h followed by a 200 µm H_2_O_2_ challenge for 6 h H_2_O_2_ treatment alone caused approximately 60% cell viability loss in mLECs (Figure 2). In contrast, treatments of ^OH^Py_2_N_2_ at concentrations of both 10 μm and 100 μm ^OH^Py_2_N_2_ protected cells from oxidative stress-induced cell damage, with the later showing the largest (90%) improvement. The cell morphology was then observed under phase-contrast microscopy. Following exposure to 200 μm H_2_O_2_ for 6 h, LECs showed a marked decrease in number concomitant to shrinkage in size and loss of adhesion. Pretreatment with different doses of ^OH^Py_2_N_2_ (10 and 100 µm) attenuated H_2_O_2_-induced cell morphological changes. These results suggest that the ^OH^Py_2_N_2_ molecule can prevent cell death induced by oxidative stress in multiple models, making its translation to multiple disease states arising from oxidative stress promising.

### ^OH^Py_2_N_2_ prevents ROS production

**FIGURE 3:**
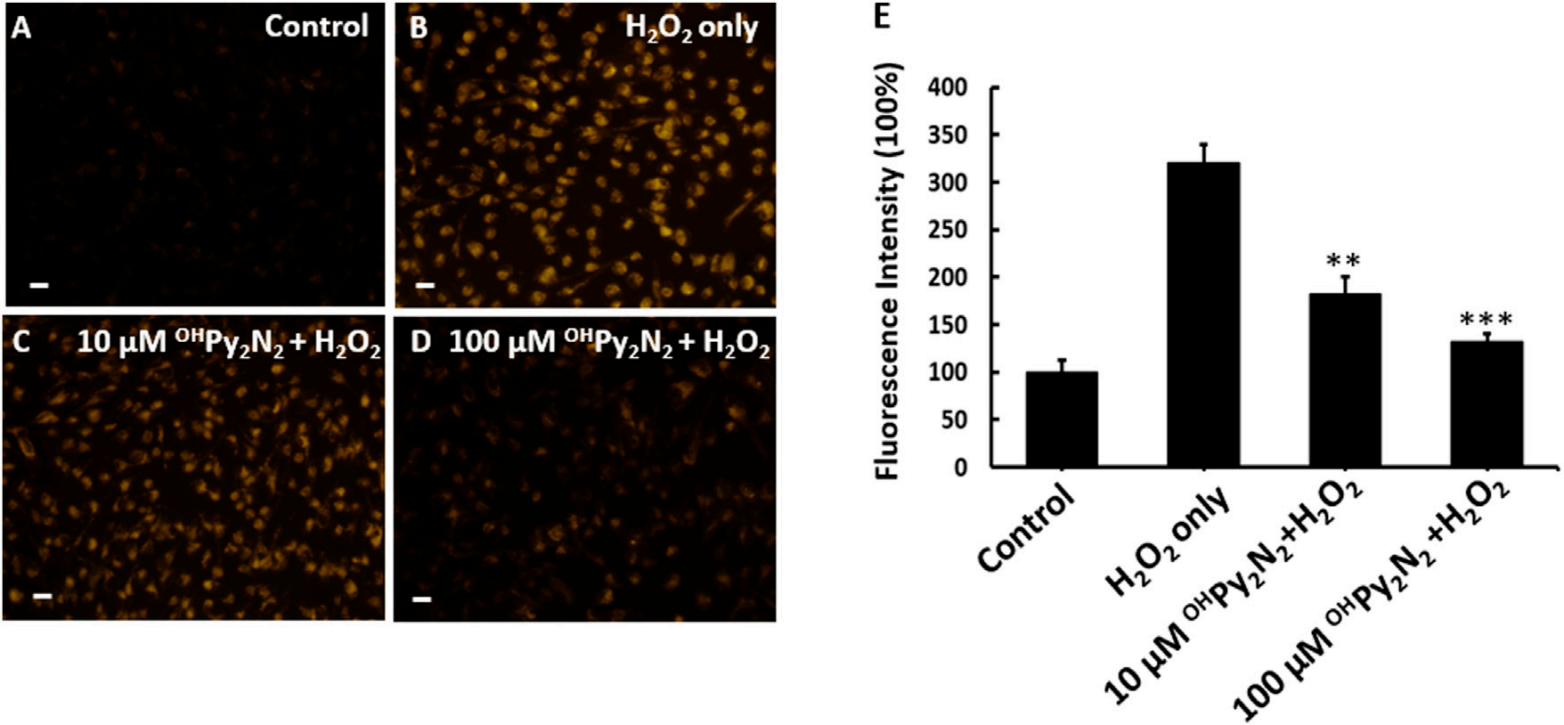
^OH^Py_2_N_2_ reduces ROS production in H_2_O_2_-treated LECs. **(A)** LECs as a control with no treatment, **(B)** 200 μM H_2_O_2_ treatment for 30 min, and **(C,D)** pretreatment with 10 or 100 μM ^OH^Py_2_N_2_ for 24 h followed by 200 μM H_2_O_2_ treatment for 30 min. **(E)** Fluorescence was detected using the probe CellROX orange reagent for all groups; fluorescence intensity quantified and represented as the mean mean ± SEM of three independent experiments, **p < 0.01, ***p < 0.01 compared with the H_2_O_2_-only group (*n* = 3). Scale bar = 50 μm.

Intracellular ROS levels were measured to determine if the protection demonstrated by ^OH^Py_2_N_2_ against H_2_O_2_ (Figure 2) induced cell death as a result of lowering the oxidative stress in the LECs. For this study, cells were preloaded with CellROX Orange followed by stimulation with H_2_O_2_ to monitor the resulting ROS sensitive fluorescent product (Figure 3). Separately, LECs were pretreated with ^OH^Py_2_N_2_ for 24 h and subsequently treated with H_2_O_2_ at 200 µm for another 30 min. As compared with non-treated control cells (Figure 3A), H_2_O_2_ treatment significantly increased the fluorescence intensity of ROS (Figure 3B). ^OH^Py_2_N_2_ treatment dose-dependently decreased the production of ROS (Figures 3C,D). Quantitative fluorescence intensities of CellROX Orange in the various groups are shown in Figure 3E to support the visual results and validate that the ^OH^Py_2_N_2_ molecule protects against ROS induced cell death by H_2_O_2_ by decreasing the oxidative stress in the LECs.

### ^OH^Py_2_N_2_ activates Nrf2 and increases glutaredoxin (Grx) expression

**FIGURE 4:**
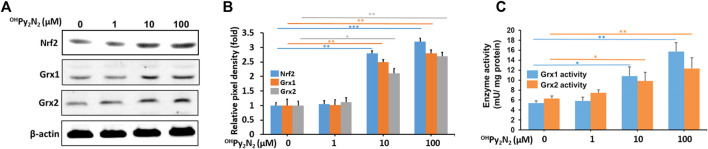
^OH^Py_2_N_2_ upregulates Nrf2, Grx1, and Grx2 in LECs.**(A)** Western blot analysis showing the increased expression of antioxidant transcription factor Nrf2 along with enzymes Grx1, and Grx2. **(B)** The relative pixel density of Nrf2, Grx1, and Grx2 over loading control. **(C)** Grx1 and Grx2 enzymatic activity increased as a result of exposure to ^OH^Py_2_N_2_. (*p < 0.05; **p < 0.01; ***p < 0.001) (*n* = 3).

Nrf2 is the central regulator of a comprehensive and protective biological antioxidant response. Activation of Nrf2 leads to the transcriptional activation of a broad range of antioxidant enzymes including NADPH dehydrogenase, heme oxygenase-1, superoxide dismutase, catalase, glutathione peroxidase (GPX), thioredoxin, and more. Our previous study has shown that ^OH^Py_2_N_2_ could dose-dependently upregulate Nrf2 in neuronal cell culture (Johnston et al., 2019). Therefore, LECs were treated with ^OH^Py_2_N_2_ at 1, 10, and 100 µm for 24 h to further investigate the effect on the Nrf2 pathway in this particular model. Western blot analysis showed a dose-dependent increase of Nrf2 protein expression (Figure 4A). This result indicates that the mode of antioxidant action (Nrf2 expression) is translated between neuronal and lens cell culture showing the portability of this molecule toward treating conditions rising from oxidative stress. As a result of this success, we next explored what mechanisms could be activated as a result of Nrf2 expression upon treatment with ^OH^Py_2_N_2_ resulting in the protective effects observed to date.

To maintain intracellular redox homeostasis, mammalian tissues including the lens have well-designed repair systems to protect protein thiols from oxidation (Holmgren, 1989; Lillig et al., 2008). The first is the GSH-dependent glutaredoxin (Grx) system, which are a group oxidoreductases that catalyze the reduction of glutathione-protein mixed disulfides. The Grx system has two major isozymes: glutaredoxin 1 (Grx1, also known as thioltransferease or TTase) and the recently discovered glutaredoxin 2 (Grx2) (Holmgren, 1976; Gladyshev et al., 2001; Lundberg et al., 2001). Previous studies suggest that enhancing Grx system may be a potential therapeutic strategy for delaying or slowing down the age-related cataracts (Xing and Lou, 2010; Kronschläger et al., 2012; Wu et al., 2014; Fan et al., 2022). In our study, we found ^OH^Py_2_N_2_ could boost activity of the Grx system. The enzymatic activity of Grx1 and Grx2 were observed to increase in cell lysates treated with ^OH^Py_2_N_2_ (1–100 μm) in a dose-dependently manner (Figure 4B) along with the protein levels of Grx1 and Grx2 (Figure 4A). From this series of studies, it can be concluded that the addition of ^OH^Py_2_N_2_ to LECs activates the translocation of Nrf2 and the expression of antioxidant enzymes, including Grx1 and Grx2 as a mechanism that results in the catalytic protection of LECs against ROS assaults. To our knowledge this is the first example of a such a low molecular weight, macrocyclic small molecule of this type, particularly a pyridinophane, that can activate cellular mechanisms of protection against oxidative stress.

### ^OH^Py_2_N_2_ demonstrates anti-ferroptotic effects and iron binding

**FIGURE 5:**
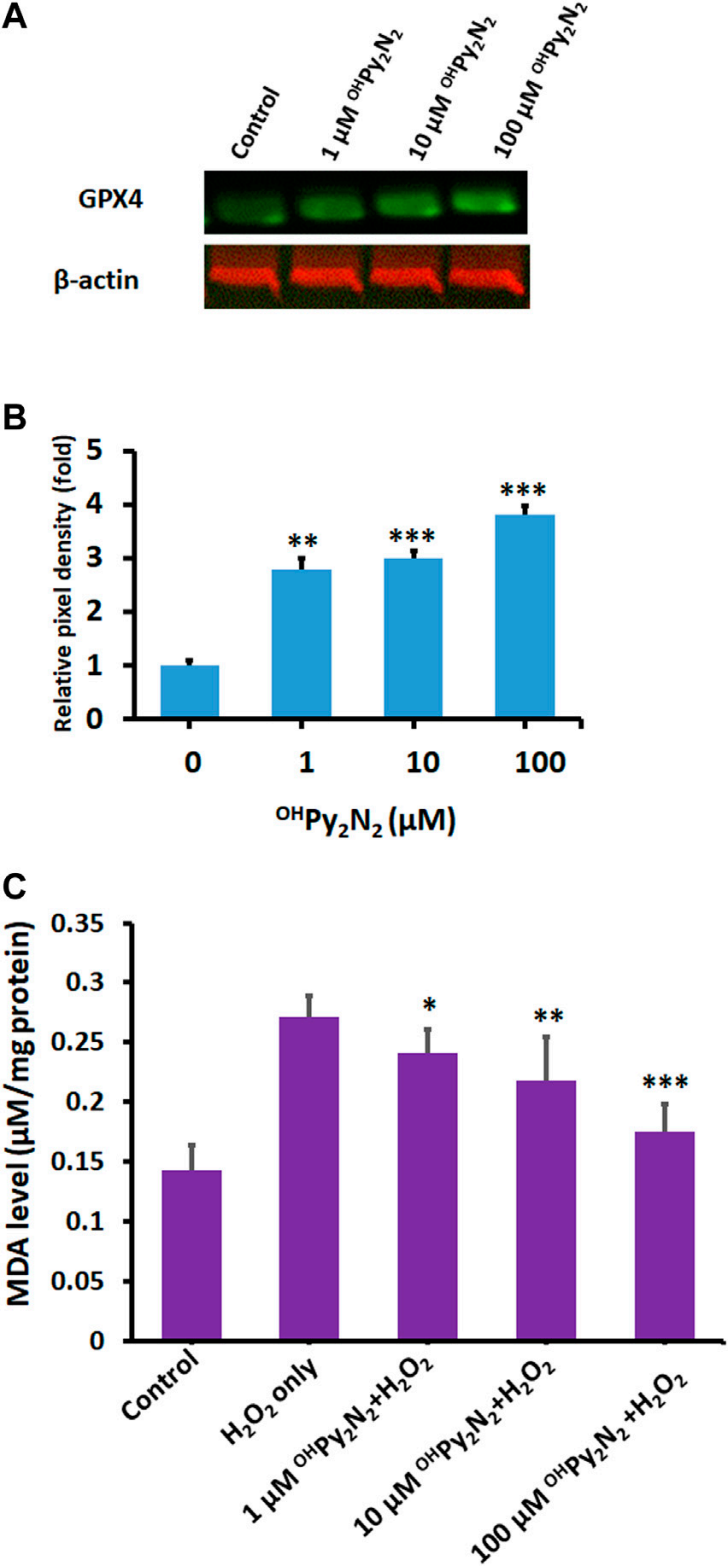
Anti-ferroptotic effects of ^OH^Py_2_N_2_ in LECs. **(A)** ^OH^Py_2_N_2_ upregulates GPX4 expression. LECs were treated with (1–100 µM) ^OH^Py_2_N_2_ for 24 h. Control cells were treated identically but without additional of ^OH^Py_2_N_2_. **(B)** The relative pixel density of GPX4 over loading control. Data presented are a typical representation of triplicate experiments. (**p < 0.01; ***p < 0.001). **(C)** ^OH^Py_2_N_2_ inhibits H_2_O_2_-induced MDA formation. Cells were incubated with or without ^OH^Py_2_N_2_ (1, 10 and 100 µM) for 24 h and then treated with 200 µm of H_2_O_2_ for 6 h. MDA level in each group was measured. (*p < 0.05; **p < 0.01; ***p < 0.001) (*n* = 3).

Ferroptosis is a newly identified form of non-apoptotic regulated cell death that is initiated by excessive iron-dependent lipid peroxidation (Jiang et al., 2021). Glutathione peroxidase (GPX4) is the key regulator of this form of cell death (Lei et al., 2022). Compounds that up-regulate GPX4 have been proposed as ferroptosis inhibitors (Chen et al., 2020; Li et al., 2020; Lei et al., 2022). In fact, inhibiting ferroptosis has the potential to be a useful therapeutic method for age-related diseases, including cataract (Wei et al., 2021). In our study, we found ^OH^Py_2_N_2_ dose-dependently upregulate GPX4 in LECs (Figures 5A,B), indicating ^OH^Py_2_N_2_, thus enhancing a natural defense mechanism against ferroptosis.

Ferroptosis is associated with two major biochemical hallmarks, lipid peroxidation and iron accumulation (Tang et al., 2021). Malondialdehyde (MDA), the final product of polyunsaturated fatty acid peroxidation, is one of the most important biomarkers of lipid peroxidation. Previous studies have shown that MDA accumulation plays a pivotal role in senile cataractogenesis (Bhuyan et al., 1986). As shown in Figure 5C, as compared with the control group, the MDA level was significantly increased after H_2_O_2_ treatment (control: 0.14 ± 0.021 vs. H_2_O_2_ alone: 0.27 ± 0.0172 nmol/mg protein, *p* < 0.001). Conversely, ^OH^Py_2_N_2_ prevented H_2_O_2_-induced MDA accumulation in a dose-dependent manner (1 µM ^OH^Py_2_N_2_: 0.24 ± 0.022 nmol/mg protein *p* < 0.05 vs. 10 µm ^OH^Py_2_N_2_: 0.21 ± 0.035 nmol/mg protein *p* < 0.01 vs. 100 µM ^OH^Py_2_N_2_: 0.17 ± 0.023 nmol/mg protein *p* < 0.001). Our data indicate that ^OH^Py_2_N_2_ effectively prevented oxidative stress-induced lipid peroxidation.

**FIGURE 6:**
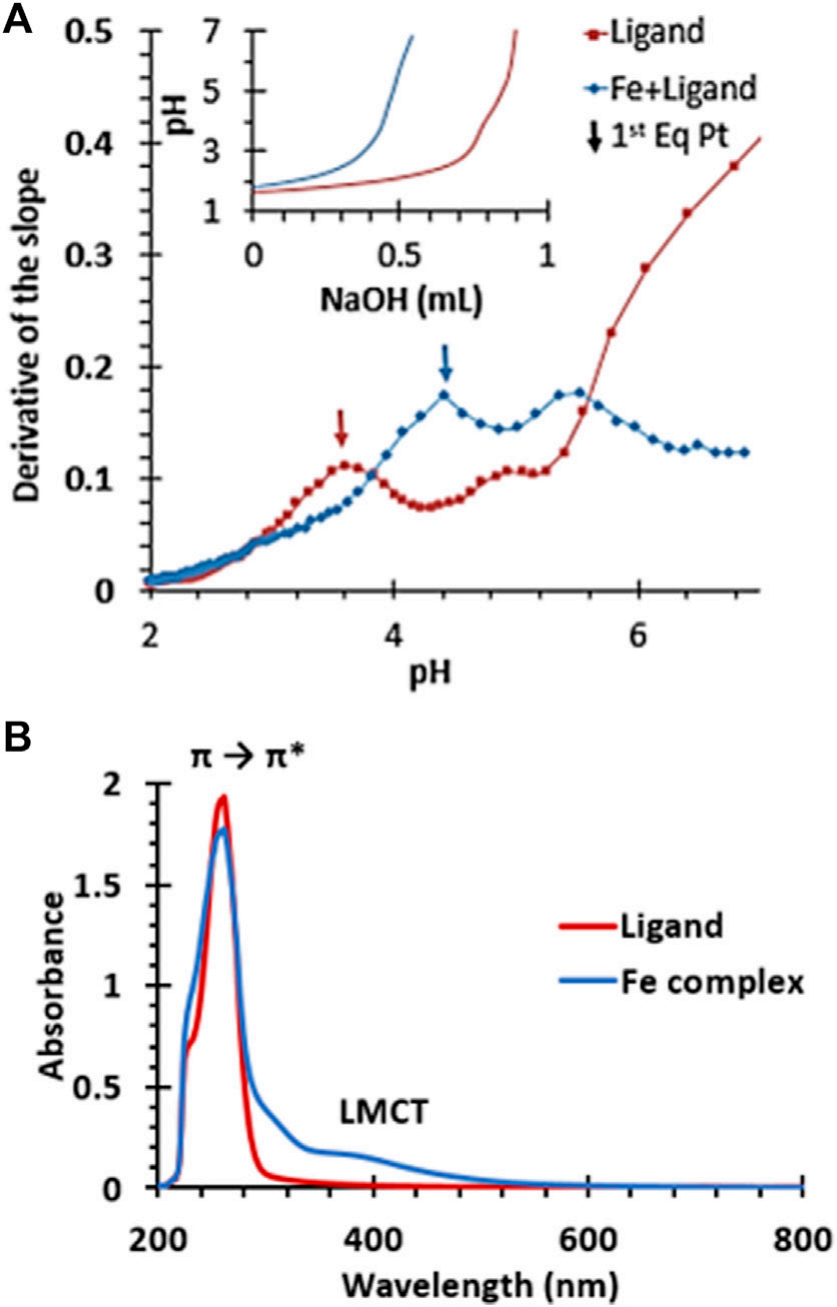
**(A)** First derivative of the potentiometric titration of ^OH^Py_2_N_2_ and the iron (II) complex. Inset shows original titration data. **(B)** UV-vis spectra of an aqueous solution of ^OH^Py_2_N_2_ (red) and **(B)** the iron (III) complex of ^OH^Py_2_N_2_.

Additionally, iron-chelating capacity is also strongly linked with anti-ferroptotic effects (Chen et al., 2020; Lei et al., 2022). The metal binding halts the Fenton reaction that leads to ferroptotic lipid peroxidation. Molecule ^OH^Py_2_N_2_ has previously been shown to bind copper (II) effectively and molecules within this pyridinophane family are well-known to interact strongly with a range of transition metal-ions including iron (II) and (III) (Brewer et al., 2018; Johnston et al., 2019; Mekhail et al., 2020; Brewer et al., 2021). Potentiometric titrations of ^OH^Py_2_N_2_ and iron (II) in aqueous solution (Figure 6) validate complexation based on the shift in the titration curve compared to the chelate molecule alone. This curve is very similar to our previous studies of similar iron (II) titrations showing complex formation around a pH of four and is consistent with a log β in the range of 12–13, which is in range or higher than metal-binding agents explored to date (Chen et al., 2020; Mekhail et al., 2020). Furthermore, addition of iron (III) to an aqueous solution of ^OH^Py_2_N_2_ resulted in a color change from pale yellow to dark yellow. UV-vis analysis of the resulting solution showed ligand to metal charge transfer bands at 313 and 390 nm (Figure 6) and a ligand based π to π* transition at 290 nm. This spectroscopy is consistent with an Fe(III) (^OH^Py_2_N_2_)Cl_2_ complex of the geometry shown in Figure 1 (Mekhail et al., 2020; Brewer et al., 2021). Together, these results serve as evidence that the ^OH^Py_2_N_2_ scaffold is a good agent for binding iron (II) and (III) and could be a contributing mechanism for antiferroptotic activity to explore in depth in the future.

### ^OH^Py_2_N_2_ treatment promotes ATP production

**FIGURE 7:**
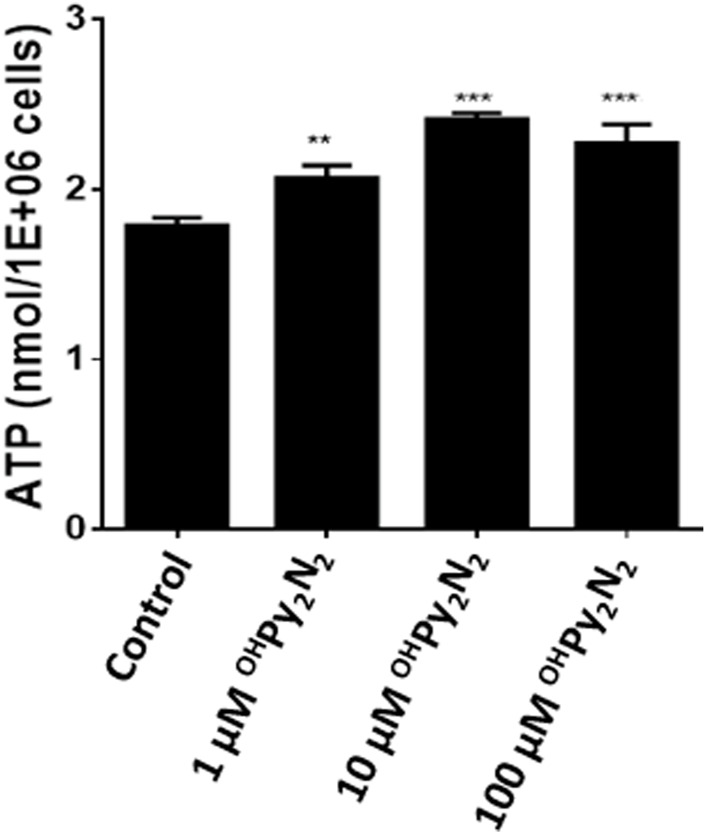
ATP production is increased with the addition of ^OH^Py_2_N_2_. LECs were treated with (1–100 µm) ^OH^Py_2_N_2_ for 24 h. Control cells were treated identically but without additional of ^OH^Py_2_N_2._ Whole cell homogenates were used for ATP measurement with an ATP assay kit (*n* = 6, **p < 0.01, ***p < 0.001).

Adenosine triphosphate (ATP) is often referred to as the “energy currency” because it is used to fill any energy needs of the cell. Reduced energy levels threaten cellular homeostasis and may trigger cataract formation (Greiner and Glonek, 2020). As a result of these foundations, the ability for ^OH^Py_2_N_2_ to elevate ATP production as a means of preventing oxidative stress induced cataract formation was explored using an established luciferase based bioluminescence assay method (Wu et al., 2014). The ^OH^Py_2_N_2_ molecule promoted a dose-dependently ATP production, suggesting that ^OH^Py_2_N_2_ may enhance mitochondrial function and improve bioenergetic function to help LECs to fight against oxidative stress (Figure 7).

### ^OH^Py_2_N_2_ protects the lens from H_2_O_2_-induced opacity

**FIGURE 8:**
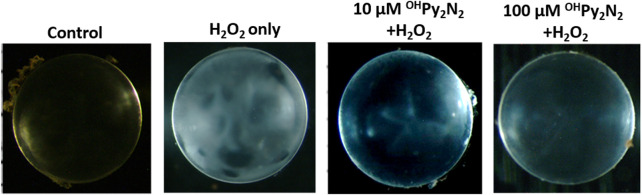
^OH^Py_2_N_2_ prevented H_2_O_2_-induced opacity in culture mouse lenses. Cultured lenses were incubated with 10–100 μm ^OH^Py_2_N_2_ for 24 h and then exposed to 200 μm H_2_O_2_ for another 6 h (*n* = 3).

Encouraged by the above positive results, we further tested if ^OH^Py_2_N_2_ treatments could prevent H_2_O_2_-induced lens opacity. Cultured lenses extracted from C57BL/6J mice were incubated with ^OH^Py_2_N_2_ (10–100 μm) for 24 h and then exposed to 200 μm H_2_O_2_ for another 6 h. As shown in Figure 8, pretreatment with 10 and 100 μm ^OH^Py_2_N_2_, followed by H_2_O_2_ induction, resulted in reduced opacities compared to cataract induction alone.

## Conclusion

**FIGURE 9:**
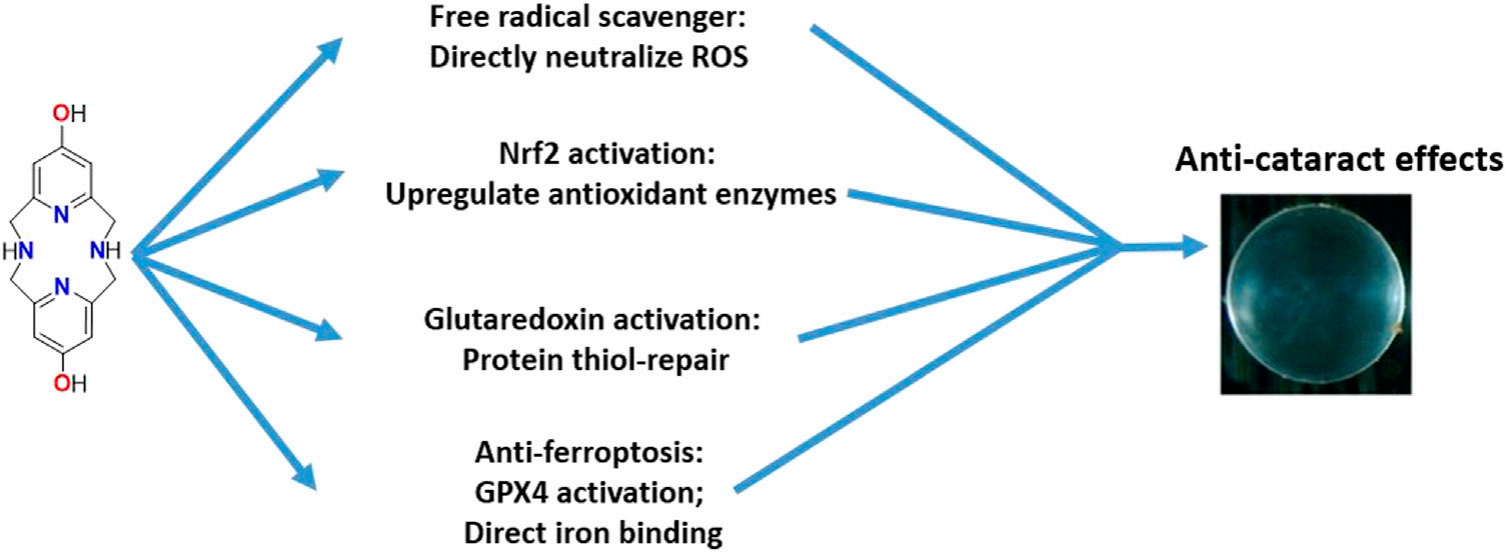
Multiple pathways are activated upon exposure to ^OH^Py_2_N_2_ that can result in protection against oxidative stress and cataract formation.

The studies show that the ability for ^OH^Py_2_N_2_ to induce protection against peroxide induced cell death over to LECs (Figure 9). Although ^OH^Py_2_N_2_ was designed to serve as a direct antioxidant that functions through radical scavenging and metal-binding, the studies described herein show that the molecule activates multiple biological pathways innate to cells that promote the cells to protect against oxidative stress. The ability for ^OH^Py_2_N_2_ to turn-on Nrf2 expression as well as Grx1 and Grx2 catalytic pathways impacted by the small molecule. The molecule also upregulates GPX4 and binds iron (II) and (III) ions, which suggests that it should be explored further for the details regarding its anti-ferroptotic abilities in more detail. The bioenergetic pathway is also upregulated by treatment with ^OH^Py_2_N_2_ as evidenced by increased levels of ATP. Lastly, cultured lenses were protected from H_2_O_2_-induced lens opacity by this multimodal small molecule, showing the ultimate potential of this water-soluble small molecule. Altogether, this work opens up several new fields of exploration that marry the organic and inorganic design of small molecules with studies that function to elucidate an understanding of their potential to impact diseases in the eye. Moreover, understanding what structural components of the ^OH^Py_2_N_2_ molecule are responsible for activating these pathways is of urgent interest. This work also leads to the need to understand at what point(s) in the immune response pathway for protecting against oxidative stress the ^OH^Py_2_N_2_ interacts, leading to the responses described herein.

## Data Availability

The original contributions presented in the study are included in the article, further inquiries can be directed to the corresponding authors.
